# Changes in microRNA expression profiles in HIV-1-transfected human cells

**DOI:** 10.1186/1742-4690-2-81

**Published:** 2005-12-28

**Authors:** Man Lung Yeung, Yamina Bennasser, Timothy G Myers, Guojian Jiang, Monsef Benkirane, Kuan-Teh Jeang

**Affiliations:** 1 Molecular Virology Section, Laboratory of Molecular Microbiology National Institute of Allergy and Infectious Diseases, National Institutes of Health Bethesda, Maryland 20892-0460, USA; 2 Microarray Research Facility, Research Technologies Branch, National Institute of Allergy and Infectious Diseases, National Institutes of Health Bethesda, Maryland 20892-8005, USA; 3 Laboratoire de Virologie Moleculaire, Institut de Genetique Humaine, CNRS UPR1142, Montpellier, France; 4 Building 4, Room 306, 9000 Rockville Pike, Bethesda, MD 20892-0460, USA

## Abstract

MicroRNAs (miRNAs) are small RNAs of 18–25 nucleotides (nt) in length that play important roles in regulating a variety of biological processes. Recent studies suggest that cellular miRNAs may serve to control the replication of viruses in cells. If such is the case, viruses might be expected to evolve the ability to modulate the expression of cellular miRNAs. To ask if expression of HIV-1 genes changes the miRNA profiles in human cells, we employed a high throughput microarray method, termed the RNA-primed Array-based Klenow Enzyme (RAKE) assay. Here, we describe the optimization of this assay to quantify the expression of miRNAs in HIV-1 transfected human cells. We report distinct differences in miRNA profiles in mock-transfected HeLa cells versus HeLa cells transfected with an infectious HIV-1 molecular clone, pNL4-3.

## Findings

MicroRNAs (miRNAs) are small RNAs of 18–25 nucleotides (nt) in length that are involved in the regulation of a variety of biological processes including developmental timing, signal transduction, apoptosis, cell proliferation and tumorigenesis [1-3]. Recent studies indicate that cellular miRNAs can variably inhibit [4] or promote [5] viral replication. Viruses, on the other hand, seem to have developed strategies which include virus-encoded RNAi suppressors [6-12] and/or virus-encoded miRNAs [13-19]. Mechanistically, a current view is that miRNAs function to silence gene expression through imperfect base-pairing with cognate transcripts. Since RNA silencing mediated by miRNA does not require perfect sequence complementarity, one miRNA can target multiply different mRNAs [20]. It is conceivable that viruses may seek to alter cellular miRNA expression in ways that benefit viral replication. Extant findings support such a notion since several viruses have been found to encode RNAi suppressors which could function to influence the cell's overall miRNA milieu [6-12].

**Figure 1:**
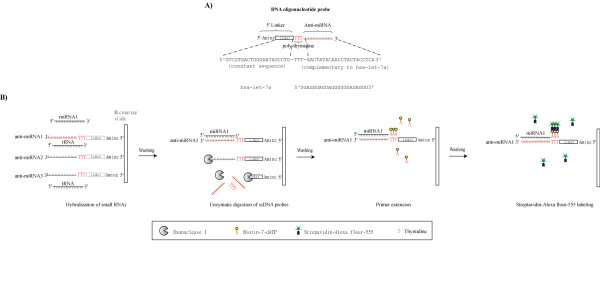
**Schematic diagram of the RAKE assay**. A) The DNA oligonucleotide probe for miRNA detection is composed of three elements. The 5' linker region contains a constant nucleotide sequence (5'GTCGTGACTGGGAATAGCCTG3') with an amine-modified 5'end which permits the probe to conjugate efficiently to the epoxy-coated microarray glass slide. The anti-miRNA region contains a sequence complementary to specific miRNA (for instance, anti-hsa-let-7a 5'AACTATACAACCTACTACCTCA3') for capturing the cognate miRNA (hsa-let-7a 5'UGAGGUAGUAGGUUGUAUAGUU3'). The poly-thymidine region acts as a template for primer extension of the hybridized miRNA using biotinylated-dATP. B) Small RNAs isolated from cells are hybridized to the microarray slide described in A. After washing, unhybridized single-stranded DNA probes (ssDNA probes) are removed by exonuclease I. Digested nucleotides are then removed leaving the hybridized miRNAs for primer extension. The poly-thymidine region now acts as a template for the hybridized miRNA to be extended using Klenow (3'→5' exo^-^) in the presence of biotinylated-dATP. Streptavidin-Alexa fluor 555 is then used to bind the biotin group permitting the fluorescent detection of hybridized miRNAs using a GenePix 4000B microarray scanner (Axon/Molecular Dynamics).

For HIV-1, it has been proposed, based on *in vitro *assays, that Tat can partially repress the processing activity of Dicer [21]. Because Dicer is involved in the maturation of cellular miRNAs, we wondered how miRNA profiles in human cells that express HIV-1 proteins might differ from counterpart cells that do not express viral genes. To ask if HIV-1 alters the expression of host miRNAs, we employed a high throughput microarray approach to quantify changes in miRNA expression. We used a platform based on the RNA-primed Array-based Klenow Enzyme (RAKE) assay. RAKE originally described by Nelson and colleagues is a microarray assay which uses on-slide enzymatic reactions and primer extension [22]. We printed specific DNA oligonucleotide probes which contain three distinct elements onto a microarray glass slide (Fig 1A). The three different elements include a 5' linker containing a constant nucleotide sequence with amine-modified 5'end for effective slide conjugation; a 3' anti-miRNA element of variable sequence which is complementary to specific miRNA; and a poly-thymidine region which allows for primer extension and labeling of hybridized miRNAs (Fig 1B). It is important to note that RAKE does not employ a sample amplification step; and the enzymes (Klenow and exonuclease I) used in this assay work in an unbiased, substrate sequence-independent way [23]. Thus, RAKE-signals faithfully reflect the true amount of miRNAs in the samples being tested. This contrasts with some conventional microarray methods which use RNA ligase to add linkers on both ends of transcripts for subsequent sample amplification. The enzyme kinetics of RNA ligase varies depending on substrate sequences; thus, amplified samples may inaccurately represent that in the original starting population [24,25]. Moreover, complete sequence complementarity of the 3'end of miRNA with the DNA oligonucleotide probe used in RAKE is absolutely required for the primer extension step. Since many mature miRNAs differ from their precursor forms and their paralogs in the 3'end sequence, this property offers a specificity advantage to RAKE over several other microarray methodologies.

**Figure 2:**
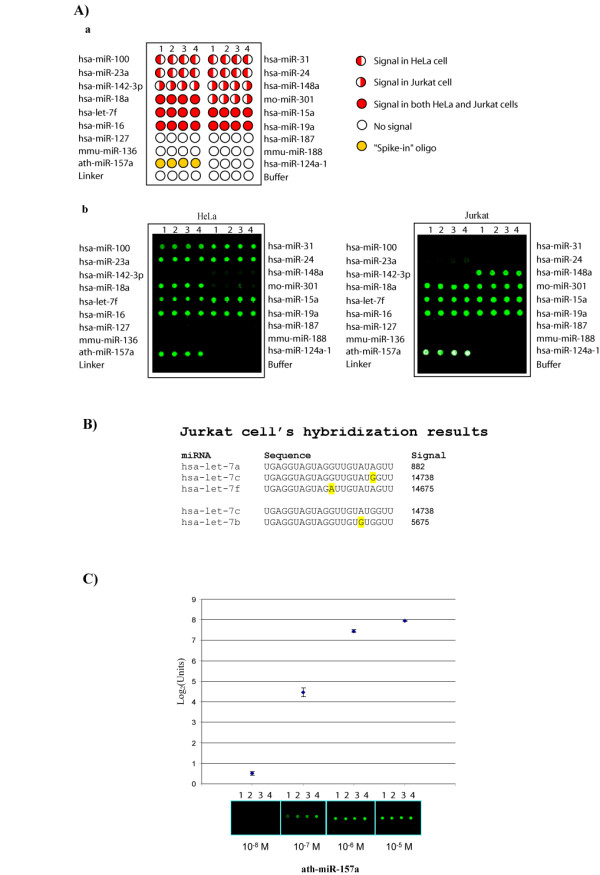
**Verification of the specificity and sensitivity of RAKE**. A) (a) A prototype small microarray designed to detect a limited number of miRNAs was first used to monitor the specificity of miRNA expression in HeLa and Jurkat cells. For purposes of verifying internal reproducibility of hybridization, each probe on the microarray slide was printed 4 times (spots 1, 2, 3 and 4 labeled at top of each column). The identity of individual probe is labeled next to the slide. Red left-hand filled circle indicates the miRNA expected to be expressed in HeLa cells. Red right-hand filled circle indicates the miRNA expected to be expressed in Jurkat cells. Red fully-filled circle indicates the miRNA expected to be present in both HeLa and Jurkat cells. Unfilled circle indicates the miRNA not expected to appear in either HeLa or Jurkat cells. Orange fully-filled circle represents "spike-in" oligos included act as positive controls to monitor successful hybridization performance. (b) We hybridized small RNAs isolated from HeLa (Left panel) and Jurkat cells (right panel) using microarray slides described in (a). Signals appear as green dots (fluorescence at 532 nm). With the exception of hsa-miR-142-3p in Jurkat cells, cell-specific signals were observed in the microarray hybridizations in patterns consistent with that expected for HeLa and Jurkat cells. 10^-5 ^M of "spike-in" oligo (ath-miR-157a) was included in the experiment as an indicator of the maximum saturating signal from RAKE (saturated signals appear in white dots). Data are presented here in raw form without further modification or normalization. B) We demonstrated the specificity of RAKE by hybridizing small RNA isolated from Jurkat cells to a subset of polymorphic miRNA (hsa-let-7 family). The names, sequences of the miRNAs and the raw signals detected from the RAKE assay are listed. hsa-let-7c and hsa-let-7f differ from hsa-let-7a in one nucleotide base (highlighted in yellow). However, the signals detected for hsa-let-7a are approximately 16 times less than that detected for hsa-let-7c and hsa-let-7f, suggesting that RAKE can distinguish a single base difference. Similarly, the signals detected for hsa-let-7c are approximately 2.5 times higher than that detected in hsa-let-7b which has only one nucleotide difference (highlighted in yellow). C) To estimate the sensitivity of the RAKE assay, different concentrations of "spike-in" oligo (ath-miR-157a) were hybridized to the small microarray described in (a). The raw data from the four different hybridization reactions (each measuring four replicated spots) are presented on the X-axis at the indicated concentrations of "spike-in" target oligo. Signal intensity of each spot (median pixel) was measured and converted into log_2 _scale. A linear range of detection can be observed when the log_2 _values are plotted against the concentration of the "spike-in" oligos between 10^-8 ^to 10^-6 ^M. An approximate minimum detectable concentration in this RAKE assay is 10^-7 ^M. Error bars represent the standard deviation of the values from the four replicated spottings of each probe.

To validate and optimize our RAKE analysis, we first printed, based on the published miRNA literature, a small number of DNA probes on glass slides. Our initial sampling set was designed to distinguish between miRNAs reported to be expression-specific for Jurkat versus HeLa cells [22] (Fig 2Aa). We wanted to verify that if we hybridized our slides with miRNAs isolated from HeLa cells, then only HeLa-specific signals would appear in our RAKE assay. Similarly, we wanted to validate the converse for Jurkat miRNAs. When we performed the assays, we indeed replicated the expected cell-specific miRNA expression patterns, with a single exception for hsa-miR-142-3p. Hsa-miR-142-3p was reported by others to be expressed in Jurkat cells, but was not detected by us in those cells (Fig 2Ab). It is unclear why hsa-mirR-142-3p was not detected in our assay, but a trivial explanation might be because there are many different lines of Jurkat cells used in various laboratories. We note that our routinely included "spike-in" oligo (ath-miR-157a), used as a control for the success of the enzymatic reaction, behaved reproducibly from experiment to experiment. We also chose a subset of polymorphic miRNA (hsa-let-7 family) in order to verify the specificity of hybridization detected by our RAKE. Using small RNAs isolated from Jurkat cells for hybridization, RAKE was able to distinguish a single nucleotide difference (hsa-let-7a from hsa-let-7c and hsa-let-7f; hsa-let-7c from hsa-let-7b), suggesting the conditions used by us are highly stringent (Fig 2B).

The sensitivity of RAKE was evaluated by hybridizing microarray slides with varying amounts of ath-miR-157a. As shown in Fig 2C, RAKE provided robust signals when challenged with as low as 10^-7 ^M of substrate, and offered linear readouts in log_2 _scale for substrates in the 10^-8 ^to 10^-6 ^M range. We defined our signal as the median of foreground spot fluorescence at 532 nm wavelength minus background (defined by surrounding pixel intensity); negative values were reset as zero.

**Figure 3:**
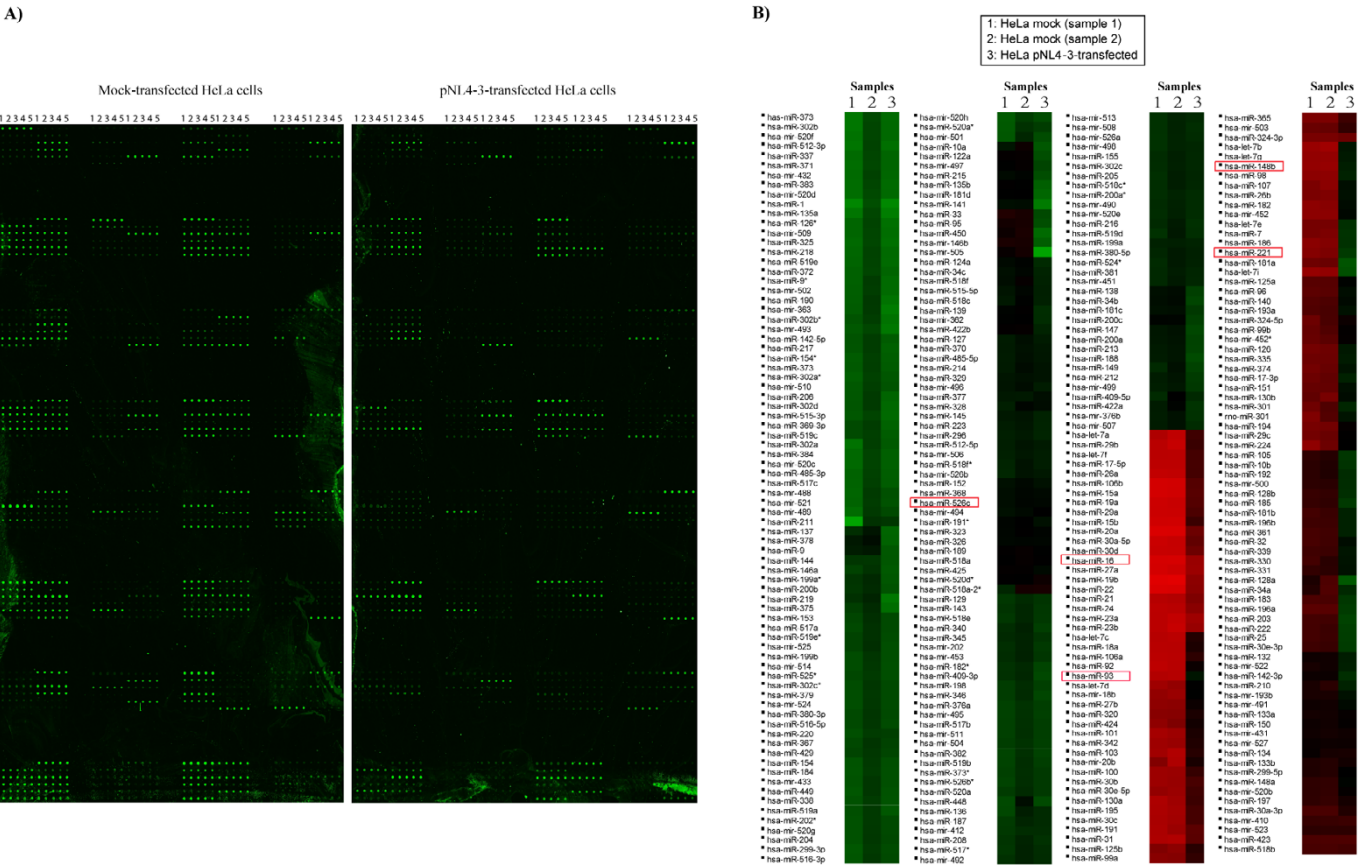
**Changes in miRNA profile after transfection of HeLa cells with HIV-1 pNL4-3**. A) Example slide readouts are shown using small RNAs isolated from mock-(left panel) and pNL4-3-transfected HeLa cells (right panel). Here, each probe was printed 5 times in a row (spots 1, 2, 3, 4 and 5 labeled at top of the arrays). Signals appear as green dots. B) Cell plot analysis of the miRNA expression profiles of mock-transfected HeLa cells (samples 1 and 2) and pNL4-3-transfected HeLa cells (sample 3). Each colored block represents the expression of one miRNA (labeled on the left) in the indicated sample. Signals acquired from the microarray are converted into color (high signal = red; low signal = black; no signal = green). Samples 1 and 2 (mock-transfected HeLa cells) show highly similar color patterns while sample 3 (pNL4-3-transfected HeLa cells) shows reduced miRNA expression (i.e. only a few blocks appear red in color). The red-boxed miRNAs were chosen for real-time PCR validation in figure 5.

**Figure 4:**
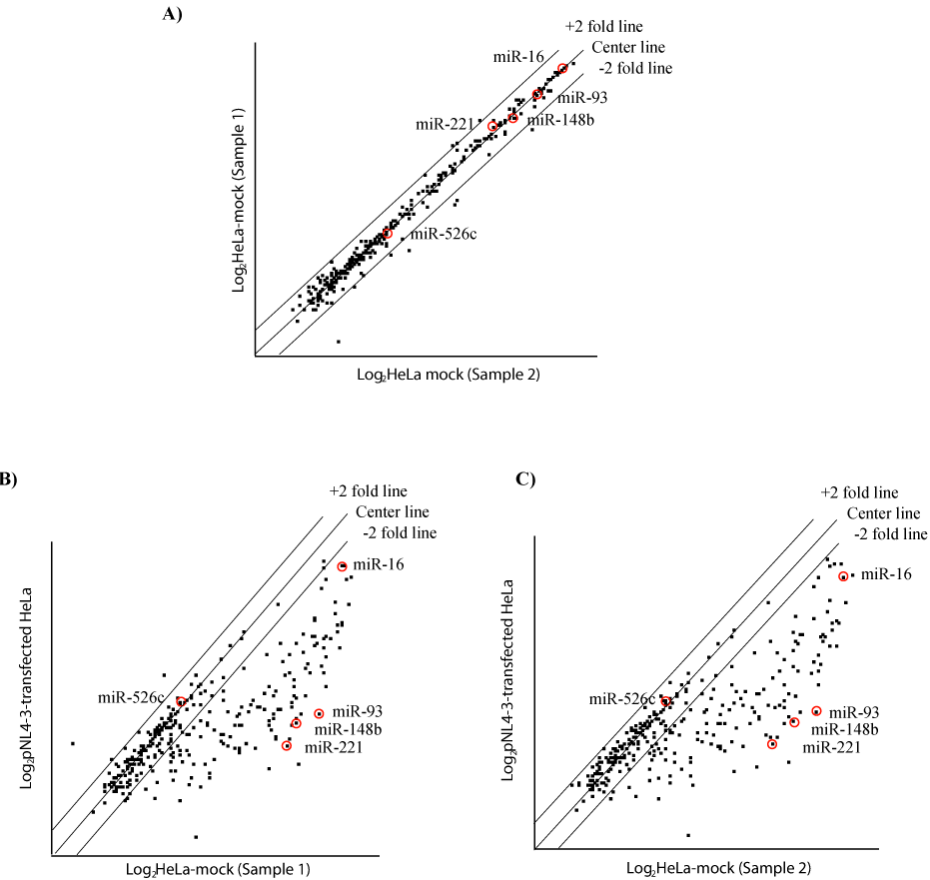
**Scatterplot analysis of the changes in miRNA expression after transfection of HeLa cells with HIV-1 clone pNL4-3**. Pairwise comparison of two mock-transfected HeLa cells (sample 1 vs. sample 2) to each other and to pNL4-3-transfected HeLa cells (sample 3) by scatterplot analysis. Spots associated with individual miRNAs were collected and converted into log_2 _scale. Each datum point represents one unique probe sequence (based on median values from 4 replicated spots from each hybridization). miRNAs with similar signal intensities from the two samples being compared line up together on a 45° diagonal line (center line). This is most clearly seen in (A), where two mock-transfected HeLa cells samples are compared to each other. In this comparison, most of the dots line up together at the center line supporting that the miRNA expression patterns of the two samples (1 and 2) are highly similar. By contrast, miRNAs with expression levels higher or lower in one sample than the other sample are expected to produce dots that deviate from the center line. The dots are allocated to positions that are above or below than the +2 fold or -2 fold line when the differences are greater than two folds. This was the case when the log_2 _values of sample 3 (pNL4-3-transfected HeLa cells) was plotted against sample 1 (B) or 2 (C). The miRNAs with reduced expression in sample 3 are allocated to positions below the -2 fold line. The red-circled miRNAs were chosen for real-time PCR validation as shown in figure 5.

After optimization of conditions in initial small scale tests, we next printed microarray slides which contained 312 individual probes based on published sequences of all-known mature human miRNAs at time of slide production. We separately hybridized individual slides with small RNAs (20 μg per slide) isolated from mock-transfected HeLa or HeLa cells transfected with infectious HIV-1 molecular clone, pNL4-3 (see Fig 3A for actual examples of typical results). The results from cell plot analysis of repeated hybridizations indicated that large numbers of miRNAs in pNL4-3-transfected HeLa cells, when compared to mock-transfected HeLa cells, were significantly downregulated (Fig 3B). Clear differences were revealed in comparisons of mock-transfected HeLa cells to pNL4-3-transfected HeLa cells using scatterplot analysis (Fig 4). Although many miRNAs were reduced in expression in the HeLa-pNL4-3 sample (e.g. ~43% of all of the miRNAs were more than two-fold downregulated), the majority of miRNAs remained unchanged, suggesting that the observed results are not due to non-specific generalized cellular toxicity. Interestingly, in our assays, miRNAs upregulated by transfected pNL4-3 were exceedingly rare. Pending further understanding of mechanisms, it is conceivable that the downregulation of mature miRNAs as detected by our RAKE assay may be due to the Dicer-suppressive effect exerted by HIV-1 Tat protein and/or TAR RNA [21,26].

**Figure 5:**
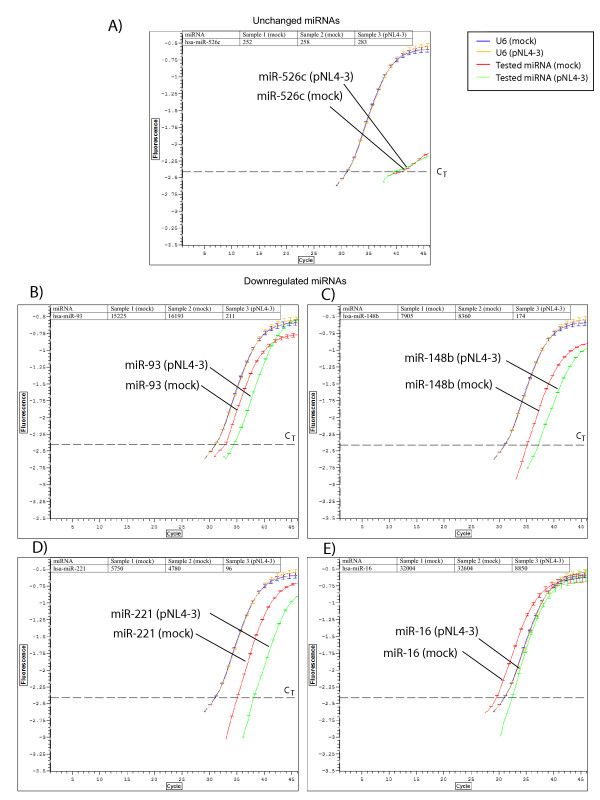
**Validation of RAKE using real-time PCR**. Fluorescence signals from each of the 45 PCR cycles were collected and converted into log_10 _values. The log_10 _fluorescence values (Y-axis) of each sample are then plotted against the PCR cycles (X-axis) to generate a sigmoid curve. C_T _(threshold-cycle; dotted line) determines the minimum PCR cycle required for the reaction to give a threshold fluorescence signal. Samples with more templates require fewer PCR cycles to reach the threshold. Comparison of the miRNA expression levels in pNL4-3- (green curve) and mock-transfected HeLa cells (red curve) are facilitated by using cellular small nuclear U6 RNA (blue curve from mock, and orange curve from pNL4-3; please note that the blue and orange control curves superimpose closely on top of each other, supporting the validity of the PCR conditions for comparing the experimental curves) and an empirically established unchanged miRNA (mi-526c; mock and pNL4-3 samples are shown in red and in green curves, respectively) as normalization references (A). Selected pNL4-3-downregulated miRNAs [miR-93 (B), miR-148b (C), miR-221 (D) and miR-16 (E)] were validated by real-time PCR. Real-time PCR curves for U6 RNA control (mock and pNL4-3) are included in all of the graphs for normalization. Signals of the selected miRNAs measured in the RAKE assay from different samples (1, 2 and 3) are presented in table form at the top of each graph.

To confirm our RAKE assays, we tested selected results using real time PCR as described by Shi and Chiang [27]. Using these assays, we checked the RAKE results in HeLa cells for four HIV-1 downregulated miRNAs (miR-93, miR-148b, miR-221 and miR-16) (Fig 5B, C, D and 5E). We used two normalization controls, a miRNA (miR-526c) whose expression was found empirically to be reproducibly unchanged in our assays, and a miRNA-unrelated small cellular RNA, the small nuclear U6 RNA (Fig 5A). Real time PCR results confirmed the findings from RAKE.

In conclusion, we describe here a rapid assay that monitors reproducible changes in cells transfected with HIV-1 infectious molecular clone, pNL4-3. We find that a dominant pattern of response in HeLa cells to pNL4-3 transfection is the downregulated expression of many miRNAs. Studies are ongoing to examine changes in miRNA expression patterns in human cells (primary and T cell lines) after infection with HIV-1.

## Competing interests

The author(s) declare that they have no competing interests.
